# Modeling and Experimental Characterization of Bonding Delaminations in Single-Element Ultrasonic Transducer

**DOI:** 10.3390/ma14092269

**Published:** 2021-04-27

**Authors:** Wenxiang Ding, Maxime Bavencoffe, Marc Lethiecq

**Affiliations:** GREMAN UMR7347, Université de Tours, CNRS, INSA-CVL, 3 Rue de la Chocolaterie, 41000 Blois, France; maxime.bavencoffe@insa-cvl.fr (M.B.); marc.lethiecq@insa-cvl.fr (M.L.)

**Keywords:** delamination, electromechanical admittance, finite element method, structural health monitoring, ultrasonic transducer

## Abstract

Ultrasonic transducers performance can be seriously deteriorated by loss of adhesion between some constitutive elements such as the active element, the backing, or the matching layer. In the present work, the influence of bonding delaminations on the performance of a single-element ultrasonic transducer, which is composed of a piezoelectric disk, a backing, and a matching layer, is studied numerically and experimentally. Based on the positions between layers, two cases, i.e., delaminations between ceramic and backing or between ceramic and matching layer, are considered. Each case involves three different types of delaminations, which are marked as delamination type (DT)-I, II, and III. DT-I, a circular shape delamination, starts from the center and expands towards the peripheric zone; DT-II, an annular shape delamination, starts from the peripheric zone and expands towards the center; DT-III is a sector shape delamination with a given angle. The numerical simulations are performed by the finite element method and the influence of delaminations on the electromechanical admittance (EMA) of the transducer is investigated. 3D printed backings and matching layers are mounted on a PZT sample to assemble delaminated single-element transducers. An impedance analyzer is used for experimental measurements. Comparison between numerical and experimental results shows a reasonable agreement making changes in EMA an interesting indicator to inform about the occurrence and severity of delaminations in a single-element ultrasonic transducer.

## 1. Introduction

Ultrasonic transducers have been widely used for applications, including medical diagnosis and therapy, non-destructive evaluation (NDE), and underwater sonar. The proper functioning of the transducer itself is a key factor in the reliability of the entire system. However, due to the misuse of operators or material degradation, defects may occur, such as breakages in cables, cracks, damaged or weakened crystals, and delamination between layers [1,2,3,4]. This can lead to inaccurate interpretations and false positives or false negatives in applications such as medical diagnostics. Therefore, it is necessary to test the characteristics of the transducer periodically during its lifetime and detect any defect that could appear before they significantly affect the performance of the system.

Traditional methods of detecting the defects in a transducer include visual inspections, electroacoustic tests, and phantom-based measurements. Visual inspection is practical for the detection of visible physical faults. Electroacoustic tests are performed in water with commercially used devices, such as FirstCall and the ProbeHunter system. Transmit–receive signals are collected and analyzed, then parameters for each element, such as the sensitivity, pulse width, center frequency, or capacitance are displayed [2,5]. The Phantom-based method evaluates the performance of the transducer based on the quality of the image collected on a tissue-mimicking material. The inspected parameters are the homogeneity, penetration depth, beam profile, axial and lateral resolution [6,7,8]. These methods are based on the transmit-receive response of the transducer performed either in a phantom or water. They are equipment-reliant, time-consuming and are often not accurate [9]. Recently, a novel method focusing on the uniformity of the in-air reverberation pattern has been proposed [10,11,12]. It is based on the measurement of ultrasonic waves traveling and reflecting through different layers inside the transducer. The changes in the reverberation pattern may indicate the occurrence of defects, but it is difficult to give a detailed interpretation of what they are caused by.

In the field of structural health monitoring (SHM), changes in the electromechanical impedance [13,14,15,16], or admittance [17,18,19,20], are often used to detect defects inside a structure; for example, cracks or debondings between different layers. The physical basis for this technique is that the electrical impedance of the sensor is not only related to the mechanical impedances of sensor itself, but also related to the health condition of the host structure and adhesive interface [21]. Unlike traditional methods, this technique is performed directly in frequency domain instead of time domain. In the continuity of our previous work [22], we propose here an original way to follow intime integrity of the transducer thanks to its electromechanical admittance (EMA).

To this end, a typical single-element ultrasonic transducer consisting of a piezoelectric disk, a backing, and a matching layer is studied numerically and experimentally. Depending on the geometric shape, three types of delaminations between different elements are considered. Their influence on the EMA is investigated and several quantitative indicators are proposed to reveal the occurrence and extent of delaminations.

## 2. Materials and Methods

### 2.1. Studied Single-Element Transducer

**Figure 1:**
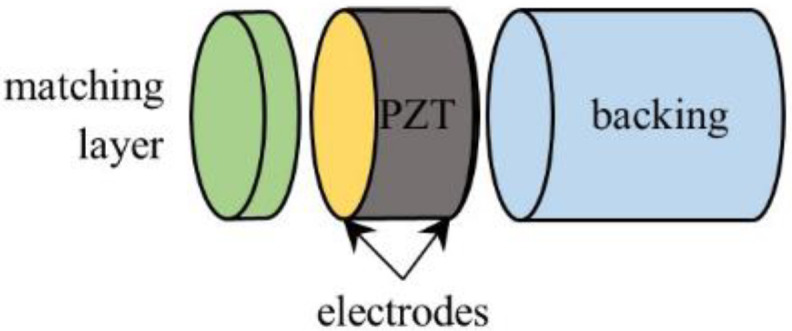
Diagram of a single-element transducer made of a matching layer, a piezoelectric disk (PZT) and a backing.

A classical single-element ultrasonic transducer (Figure 1) is composed of a piezoelectric disk, typically a PZT ceramic, with an electrode on each of its surfaces and polarized along the thickness direction, a thick backing, and one or several matching layers. The backing is added on the rear face of the ceramic disk to allow acoustic energy to flow in, thus inducing damping of the resonance. The sensitivity is therefore decreased but a higher axial resolution is achieved. On the front face, one or serval matching layers are added to match the large discrepancy in acoustic impedance between the piezoelectric ceramic (~33 MRayl) and the propagation medium (~1.5 MRayl). Therefore, more acoustic energy can flow towards the medium and the sensitivity is improved as well as the axial resolution.

Here, the PZT sample consists of a soft Ferroperm Piezoceramics PZ27 disk from Meggitt A/S Kvistgaard, Denmark with an electrode on each of its surfaces and polarized along the thickness direction. The PZT sample is 1.13 mm-thick (*t*) and has a diameter (*ϕ*) of 16 mm. Its resonance frequency (*f_r_*) is around 1.71 MHz. Material properties of PZ27 are given in Table 1 [22,23], where *ρ* is the density, $c_{ij}^{D}$ are the elastic constants, $h_{kj}$ are piezoelectric constants, $\beta_{kk}^{S}$ are the dielectric constants, $\delta_{m}$ and $\delta_{e}$ are the mechanical and dielectric loss factors, and *Z* is the acoustic impedance.

The backing is fabricated by a 3D printer using polylactic acid (PLA [24]) filament. Based on DeSilets’s optimization theory [25], the ideal acoustic impedance for the matching layer is given as

(1) $$Z_{m}=\sqrt[{3}]{Z_{p}Z_{f}^{2}}$$

where *Z_p_*, *Z_f_*, and *Z_m_* are the acoustic impedance of the piezoceramic disk, the front loading, and the matching layer, respectively.

Then, for a PZ27 disk radiating in water, the ideal value of acoustic impedance for the matching layer is 4.23 MRayl. Here this matching layer is 3D printed using a copper-filled PLA material with an acoustic impedance of 4.09 MRayl, which is very close to the calculated one by (1) (discrepancy of less than 4%). The thickness of the quarter-wave matching layer is given as ${t=\lambda }_{L}/{4=c}_{L}/{4f}_{r}=0.18$ mm, where $\lambda_{L}$ and $c_{L}$ are the wavelength and velocity of the longitudinal (*L*) waves in the matching layer. Components are bonded by an acoustic coupler phenyl salicylate (salol [26]) that is reusable since it melts at 42 °C. Thickness and material properties of passive elements are summarized in Table 2, where $c_{T}$ is the transverse wave velocity. Their diameters are the same as that of the ceramic.

### 2.2. Electromechanical Admittance

**Figure 2:**
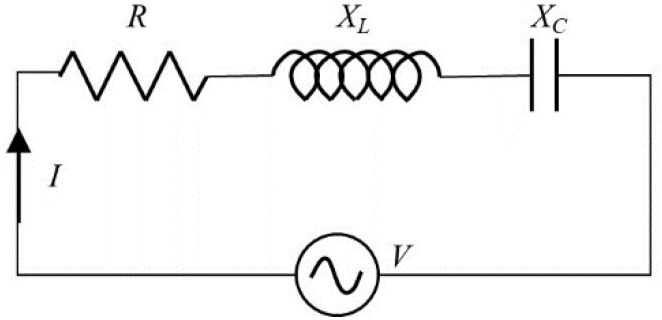
Diagram of LCR circuit (the resistance *R*, the inductance *L*, and the capacitance *C*).

In the electrical circuit theory, the electrical admittance *Y* refers to how easily a circuit will allow a current to flow inside. It is the reciprocal of electrical impedance *Z*. An electrical circuit composed of the resistance (*R*), inductive reactance (*X_L_*), and capacitive reactance (*X_C_*) is depicted in Figure 2. It is operated under an alternating voltage source. The complex admittance can be described by a real part (conductance *G*) and an imaginary part (susceptance *B*). Relationships between these terms can be expressed as

(2) $$Y\left(\omega \right)=\frac{I\left(\omega \right)}{V\left(\omega \right)}=\frac{1}{Z\left(\omega \right)}=\frac{1}{R\left(\omega \right)+iX\left(\omega \right)}=G\left(\omega \right)+iB\left(\omega \right)$$

Considering the backing and matching layer as a double-side mechanical loading on the ceramic disk, the structural changes in these passive elements or in the adhesive layers are reflected in its electrical admittance or so-called electromechanical admittance (EMA), owing to the mechanical loading effect of the passive elements. The health state of the transducer is closely related to the damping effect of the resonance characteristics; thus, the conductance *G*(*ω*) is used to track structural changes in the system.

Traditionally, the statistical based metrics, such as root mean square deviation (RMSD) and damage index (DI), have been widely employed to characterize the changes in EMA [14,27,28,29]. The RMSD index is defined as

(3) $$\mathrm{RMSD}\left(\%\right)=\sqrt{\frac{\sum_{i=1}^{i=N}{\left(G\left(\omega_{i}\right){-G}_{0}\left(\omega_{i}\right)\right)}^{2}}{\sum_{i=1}^{i=N}{\left(G_{0}\left(\omega_{i}\right)\right)}^{2}}}\times 100$$

where *ω* is the angular frequency, *N* indicates the number of points, *G*(*ω*) is the state at any time, and *G*_0_(*ω*) is the reference state.

Damage index is defined as

(4) $$\mathrm{DI}=1-corr\left(G\left(\omega \right){,G}_{0}\left(\omega \right)\right)$$

where $corr\left(G\left(\omega \right){,G}_{0}\left(\omega \right)\right)$ presents the correlation coefficient between the two states.

If we consider a specific vibration mode, its resonance amplitude (*A_R_*) and bandwidth (*BW_R_*) can also be used to quantify the changes. As we know, conductance reaches its maximum value *G*_max_, i.e., *A_R_*, at the resonance frequency *f_r_*. The frequencies corresponding to $G_{\max}/\sqrt{2}$ are marked as *f_1_* and *f_2_*, then the −3dB bandwidth (${BW}_{R}^{-3dB}$) is given by

(5) $${BW}_{R}^{-3dB}=\left|f_{2}-f_{1}\right|$$

## 3. Finite Element Modeling

The ability of ultrasonic transducers to convert the electric energy into mechanical form and vice versa is based on the piezoelectric effect of the material. Relationship between mechanical and electric quantities is the basis for the derivation of the finite element formulation [30,31] and it is defined as

(6) $$\begin{array}{c} T=c^{E}S-e^{\prime }E\\ {D=eS+\varepsilon }^{S}E \end{array}$$

where *S, T, E,* and *D* are respectively components of the strain, stress, electric field and electric displacement. *c^E^* is the elastic stiffness tensor, *e* is the piezoelectric coupling tensor, and $\varepsilon^{S}$ is the permittivity tensor. The apostrophe denotes matrix transpose.

For the piezoceramic disk, loss factors are added to the elastic and dielectric constants as [32,33]

(7) $$\begin{array}{c} c_{ij}^{D*}{=c}_{ij}^{D}\left({1+j\delta }_{m}\right)\\ \beta_{ij}^{S*}{=\beta }_{ij}^{S}\left({1+j\delta }_{e}\right) \end{array}$$

For passive elements, the loss factors are added to their elastic modulus *E* as [34]

(8) $$E^{*}=E\left({1+j\delta }_{m}\right)$$

**Figure 3:**
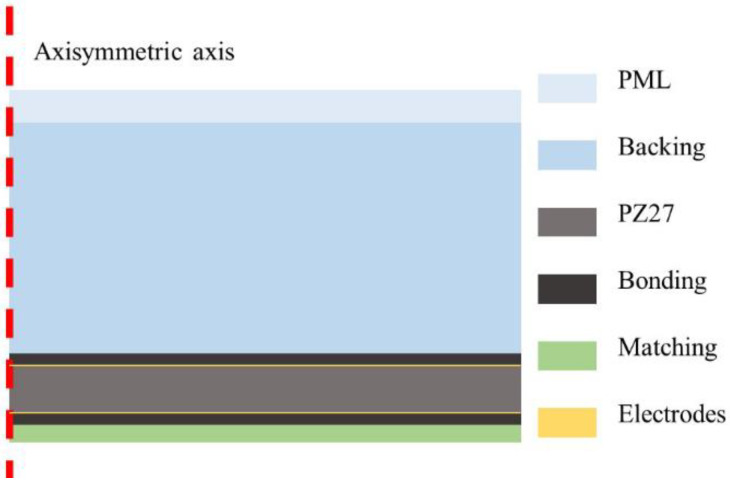
2D axisymmetric FE model of an intact single-element ultrasonic transducer.

FE simulations are developed using the commercial FE software COMSOL Multiphysics^®^ version 5.4, COMSOLAB, Stockholm, Sweden. Figure 3 shows the FE model of an intact single-element ultrasonic transducer. The backing is always much thicker than the piezoceramic disk to ensure that no reflected waves come back. To reduce the computational cost of the entire structure, a perfectly matched layer (PML) [35,36,37] is added at the rear of the backing. Chosen parameters give this polynomial stretching type PML a maximum of 109 dB attenuation for normal incidence. All simulations are developed in vacuum instead of air, because the loading effect of air on the transducer vibration is negligible [38].

Depending on the type of delamination simulated, a 2D or a 3D model is considered. The 2D model is an axisymmetric one. It is meshed with rectangular quadratic Lagrange elements of 9 nodes and a standard of 8 elements per wavelength is adopted to ensure an adequate accuracy [39]. As a result, an intact structure is shaped with a total of 5408 elements and the average element quality is 1. In the case of a 3D model, the same mesh criteria are applied on the cross section first, then, the meshed surface is swept along the axisymmetric axis. As a result, an intact structure is shaped with 79,488 hexahedral elements and 384 prism elements and the average element quality is 0.93. In both cases, a free mechanical boundary condition is applied to each end of the structure. An AC-electrical voltage source *V*(*ω*) is modeled between the electrodes and then a frequency domain analysis is performed. The frequency varies from 3 kHz to 2.4 MHz with a 3 kHz incremental step. The admittance is obtained as

(9) $$Y\left(\omega \right)=\frac{I\left(\omega \right)}{V\left(\omega \right)}$$

where *I*(*ω*) is the current flowing inside the ceramic.

### 3.1. Delamination Types

**Figure 4:**
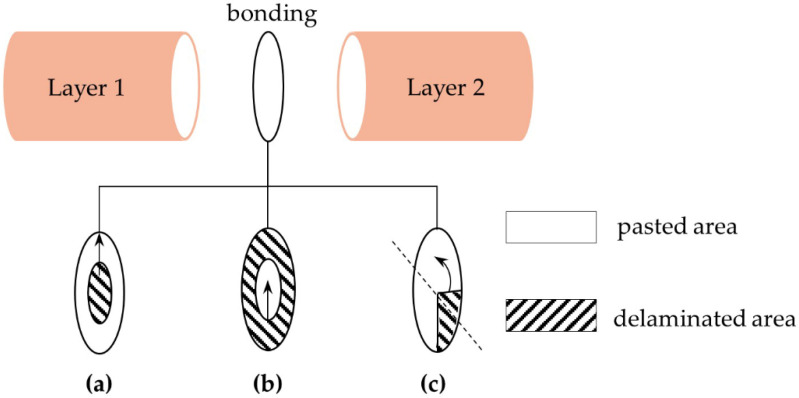
Diagram of the three delaminations types. (**a**) DT-I; (**b**) DT-II; (**c**) DT-III.

Two main cases of delaminations are studied in this work. In the first case, delaminations arise in the bonding layer between ceramic and backing, while they arise in the bonding layer between ceramic and matching layer in the second case. For each situation, three delaminations types (DT) are studied that are named respectively as DT-I (Figure 4a), a circular shape delamination, starts from the center and expands towards the peripheric zone, DT-II (Figure 4b), an annular shape delamination, starts from the peripheric zone and expands towards the center, and DT-III (Figure 4c) is a sector shape delamination with a given angle. The arrows indicate the expansion direction of the delamination.

Since the three types have different symmetric features, the first two types, i.e., DT-I and DT-II, have been studied through 2D axisymmetric modeling. However, for the DT-III, a three-dimensional (3D) model is required, using a symmetry boundary condition as indicated by the dotted line in Figure 4c.

Simulations are performed with the delamination ratio *η* varying from 0% to 100% with a step of 6.25% and *η* is defined as

(10) $$\eta =\frac{\mathrm{delaminated}\;\mathrm{area}}{\mathrm{cross}\text{-}\mathrm{sectional}\;\mathrm{area}}\times 100$$

### 3.2. Case I: Delaminations Between Ceramic and Backing

**Figure 5:**
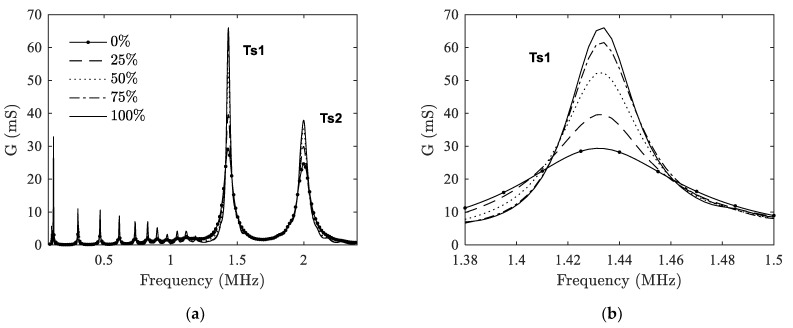
Case I: Influence of DT-I delamination on the electromechanical admittance. (**a**) frequency range 0–2.4 MHz; (**b**) peak at the first split thickness mode Ts1.

In this part, the influence of delaminations between ceramic and backing on EMA is studied. FE simulations are carried out for various delamination ratios and the results are shown in Figure 5. Since the trend of three types of delaminations are similar, DT-I type is taken as an example here. From Figure 5a we can see that the radial modes lie in the lower frequency range (<1.1 MHz), while the thickness mode lies in the higher frequency range. The thickness mode of the transducer has split into two peaks, one is at around 1.4 MHz (Ts1) and the other one is at around 2 MHz (Ts2). This is caused by the quarter-wavelength thickness matching layer in front of the ceramic when the transducer is surrounded by air.

Compared to radial harmonics, changes in the resonance peaks of thickness modes are more obvious as *η* changes. To better illustrate the changes, the peak at Ts1 is taken as an example and has been magnified as shown in Figure 5b. We can see that as *η* increases, the resonance peaks become thinner and higher. This is due to the fact that as delamination ratio increases, the damping effect of backing is reduced, leading to the increase of resonance peaks. This phenomenon appears not only on the thickness modes but also on the radial harmonics, although they are not as obvious as the former ones.

**Figure 6:**
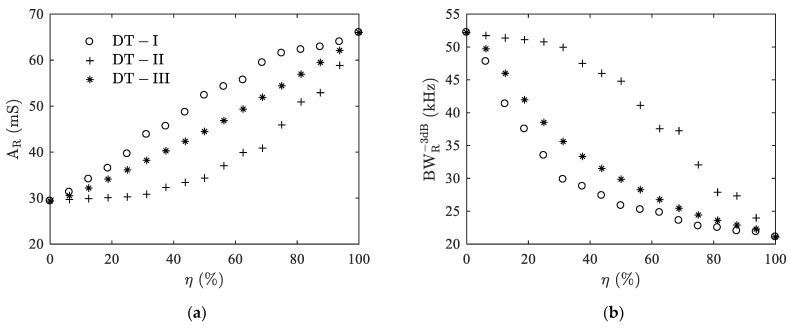
Case I: Variations of resonance amplitude (**a**) and −3 dB bandwidth (**b**) with delamination ratio *η* for the first split thickness mode Ts1.

Figure 6 shows the delamination indicators extracted from the peak Ts1 of the three types of delaminations, i.e., *A_R_* and ${BW}_{R}^{-3dB}$, versus the delamination ratio *η*. Resonance amplitude *A_R_* increases but the bandwidth ${BW}_{R}^{-3dB}$ decreases with the increasing *η*. On the other hand, it can be observed that the three delamination types show different variation patterns. The pattern of DT-III type is between those of the other two. In terms of the damaging influence of delamination on the performance of the ultrasonic transducer, such as the influence on the bandwidth, DT-I delamination makes the most, DT-III less, and DT-II even less. For example, a half delamination of the adhesive layer gives a decrease of ${BW}_{R}^{-3dB}$ from 52.2 kHz to 25.8 kHz (50.6%) for DT-I, 29.9 kHz (42.7%) for DT-III, and 44.8 kHz (14.2%) for DT-II.

### 3.3. Case II: Delaminations Between Ceramic and Matching Layer

**Figure 7:**
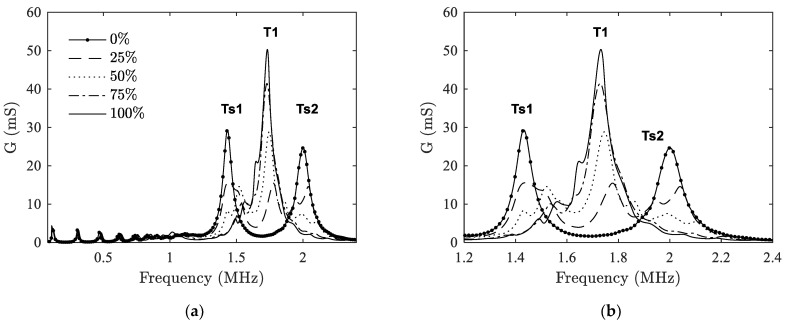
Case II: Influence of DT-I delamination on the electromechanical admittance. (**a**) frequency range 0–2.4 MHz; (**b**) peaks around the thickness mode.

A similar analysis is made for the case when delamination happens between ceramic and a matching layer. Figure 7 shows the effect of DT-I delamination on the conductance. The resonance peaks around thickness mode (T1) are also magnified for better illustration. It can be observed that the phenomenon shown here differs from that in case I. With the expansion of the delaminated area, the peaks of the two split thickness modes, i.e., Ts1 and Ts2, start to decline, while a peak in the middle position (at around *f_r_* = 1.7 MHz) gradually appears and rises up. It seems that the two split peaks merge gradually towards the original T1 mode in the middle. This is because the matching layer is losing its function as *η* increases.

**Figure 8:**
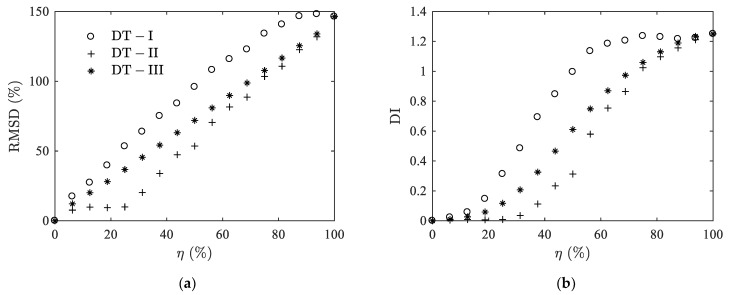
Case II: Variations of RMSD (**a**) and DI (**b**) with delamination ratio *η* for the thickness mode.

In this case, we can no longer use the delamination indicators *A_R_* and ${BW}_{R}^{-3dB}$ since they are only suitable for the characterization of a specific peak. Nevertheless, the statistical based metrics RMSD and DI are still useful: Figure 8 shows the results of the calculation of RMSD and DI in the frequency range from 1 MHz to 2.4 MHz. From an intact state to a complete delaminated state, RMSD and DI increase monotonically up to 150% and 1.25, respectively. The variation patterns of the three types still shows differences, in which the one of DT-III delamination is again in the middle of the other two.

In summary, EMA measurements can be reversely exploited to predict and reveal the occurrence of delaminations. The location of the delamination can be distinguished by whether the two split thickness peaks have merged. If the delamination happens between ceramic and backing, the severity can be quantitatively evaluated through the variation of resonance amplitude and bandwidth of the first split thickness mode Ts1. If the delamination happens between ceramic and matching layer, the severity can be evaluated through the variation of the values of RMSD or DI over the frequency range covering two split thickness modes. Discrimination of delamination types is made possible by two or more measurements at different times.

## 4. Experimental Validation

### 4.1. Experiment Setup

**Figure 9:**
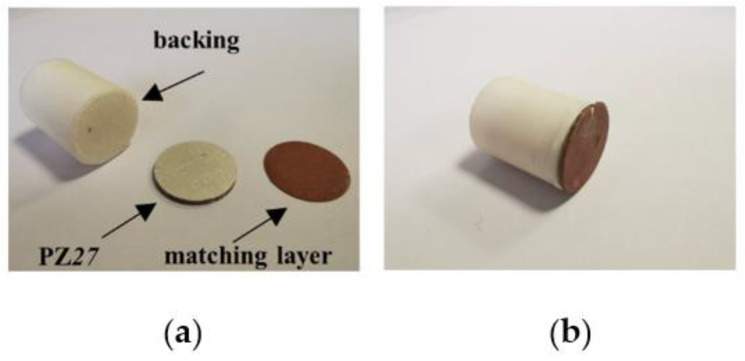
Transducer components. (**a**) a piezoceramic disk PZ27, a PLA backing and a copper-filled PLA matching layer; (**b**) an intact mounted single-element transducer.

**Figure 10:**
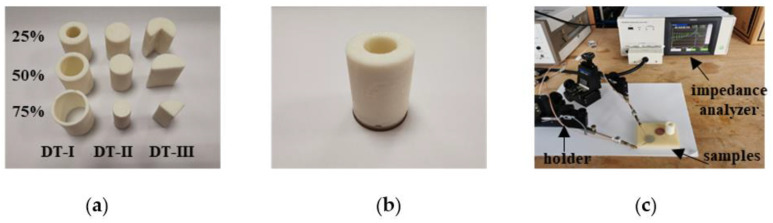
Samples and measurement setup. (**a**) nine 3D printed PLA backings; (**b**) a sample during the measurements; (**c**) experiment setup.

As mentioned in Section 2, the single-element ultrasonic transducer is realized with a PZ27 ceramic disk, a 3D printed backing and a 3D printed matching layer. These components are glued by salol, as shown in Figure 9. Geometry and materials properties have been listed in Table 1 and Table 2. The thickness of the matching layer (around 180 μm) is too thin for the 3D printer. Thus, a matching layer with a thickness of 500 μm is printed first then it is polished with a Jean Wirtz Buehler Phoenix 1000 machine to get the desired thickness. To simulate delaminations between ceramic and backing, nine backings (Figure 10a) corresponding to DT-I to -III with *η* values from 25% to 75% are printed and mounted one by one (for example Figure 10b shows the DT-I delaminated case at *η* = 25%). As for the delaminations between ceramic and matching layer, only DT-III type is realized by cutting the matching layer to its three quarter, half, and one quarter sequentially. The admittance curves are measured by the HIOKI IM3570 [40] impedance analyzer at a frequency resolution of 3 kHz in the frequency range from 3 kHz to 2.4 MHz. Figure 10c presents the experimental setup.

### 4.2. Comparison Between Experiment and FE modeling

**Figure 11:**
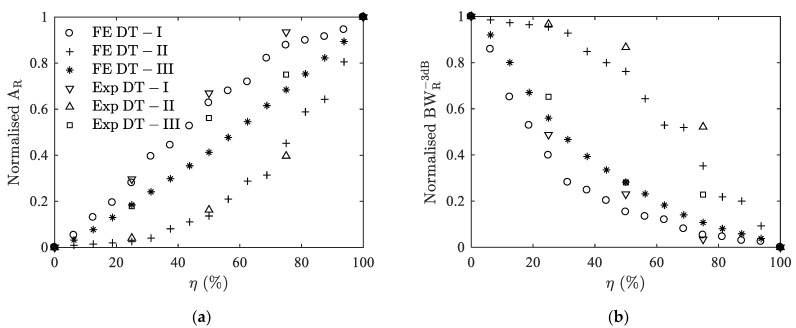
Comparison between results from FE modeling (FE) and experiments (Exp) for case I. (**a**) normalized *A_R_* versus *η*; (**b**) normalized ${BW}_{R}^{-3dB}$ versus *η*.

By mounting different delaminated backings (Figure 10a), the admittance curves are measured and collected. Figure 11 shows the variations of normalized *A_R_* and ${BW}_{R}^{-3dB}$ with delamination ratio *η*, extracted from the first split thickness mode peak Ts1. A good agreement is observed between numerical and experimental results. The evolution of the delamination indicators *A_R_* and ${BW}_{R}^{-3dB}$ coincides well and the difference between DT-I to DT-III is also confirmed by experimental results. The maximum discrepancy between experimental and numerical predicated values of *A_R_* (Table 3) are 5.7% for DT-I, 5.5% for DT-II, and 14.9% for DT-III delamination. The maximum discrepancies of ${BW}_{R}^{-3dB}$ (Table 4) are 8.9%, 16.9% and 12.1%, respectively, for these three types of delaminations.

**Figure 12:**
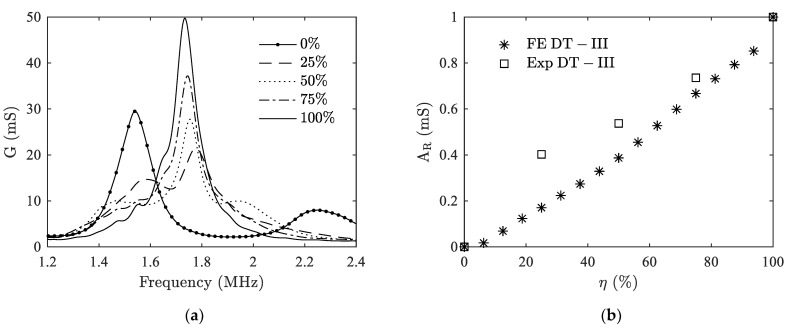
Comparison between results from FE modeling (FE) and experiments (Exp) for case II. (**a**) conductance versus frequency; (**b**) RMSD versus *η*.

As for the delamination between ceramic and matching layer, the experimental result is shown in Figure 12a. We can see that with the increase of *η*, the peaks of the two split thickness modes start to decline, while the peak at the original location of thickness mode gradually appears and rises up, leading to the increase of RMSD. The comparison between the RMSD of experiment and the FE model is shown in Figure 12b. A monotonic increasing trend is verified. Since the matching layer is very thin, it is very sensitive to the changes made including the bonding condition. Therefore, a maximum discrepancy of 37.6% is observed between the experimental result and the numerical one.

## 5. Conclusions

Based on the finite element (FE) method, a systematic study of the influence of bonding delaminations on the electrical admittance—referred to as the EMA—of a single-element ultrasonic transducer is presented. Three different types of delaminations between ceramic and backing or between ceramic and matching layer are considered. Numerical results show that changes in the EMA versus frequency curves are observed as delaminated area increases. Conversely, EMA can be exploited to detect defects inside. For the delaminations between ceramic and backing, the resonance amplitude *A_R_* and bandwidth ${BW}_{R}^{-3dB}$ of the first split thickness mode are recommended as quantitative delamination indicators. The maximum discrepancies between experimental and numerical predicated values of *A_R_* and ${BW}_{R}^{-3dB}$ are 14.9% and 16.9%, respectively. For the delaminations between ceramic and matching layer, RMSD and DI are recommended. A maximum discrepancy in RMSD of 37.6% is observed. Results obtained by the FE method and experiments are in reasonable agreement.

In summary, EMA can help to detect, locate, and characterize defects in a single-element ultrasonic transducer by qualitative and quantitative assessments during its lifetime. Future work will extend this method to multi-element ultrasonic transducers.

## Figures and Tables

**Table 1:** Geometry and material properties of the PZT sample.

*ϕ* (mm)	*t* (mm)	*ρ* (kg/m^3^)	$c_{11}^{D}$ (GPa)	$c_{12}^{D}$ (GPa)	$c_{13}^{D}$ (GPa)	$c_{33}^{D}$ (GPa)	$c_{44}^{D}$ (GPa)
16	1.13	7879.5	148.6	106.1	87.5	144.4	36.6
$h_{31}$ (GV/m)	$h_{33}$ (GV/m)	$h_{15}$ (GV/m)	$\beta_{11}^{S}$ (Gm/F)	$\beta_{33}^{S}$ (Gm/F)	$\delta_{m}$ (%)	$\delta_{e}$ (%)	*Z* (MRayl)
−0.38	1.98	1.16	9.99	0.12	0.45	0.27	33.7

**Table 2:** Geometry and material properties of the passive elements.

Components	*ϕ* (mm)	*t* (mm)	*ρ* (kg/m^3^)	$c_{L}$ (m/s)	$c_{T}$ (m/s)	$\delta_{m}$ (%)	*Z* (MRayl)
Backing	16	20	1135	1738	952.2	5	1.97
Matching	16	0.18	3284	1246	740.2	5	4.09
Bonding	16	0.002	1250	2369	994	1	2.96

**Table 3:** Discrepancy of resonance amplitude of the first split thickness mode depending on the delamination ratio (*η*).

*η* (%)	DT-I (%)	DT-II (%)	DT-III (%)
25	1.7	1.6	0.6
50	4.2	2.7	14.9
75	5.7	5.5	6.6

**Table 4:** Discrepancy of -3dB bandwidth of the first split thickness mode depending on the delamination ratio (*η*).

*η* (%)	DT-I (%)	DT-II (%)	DT-III (%)
25	8.9	1.3	9.2
50	7.7	10.4	0.1
75	1.8	16.9	12.1

## Data Availability

The data presented in this study are available on request from the corresponding author. The data are not publicly available due to privacy.
